# 5-Chloro-2-(phenyl­diazen­yl)pyridine

**DOI:** 10.1107/S1600536811047556

**Published:** 2011-11-12

**Authors:** Steffen Thies, Christian Näther, Rainer Herges

**Affiliations:** a Institut für Organische Chemie, Universität Kiel, Otto-Hahn-Platz 4, 24118 Kiel, Germany; b Institut für Anorganische Chemie, Universität Kiel, Otto-Hahn-Platz 6/7, 24118 Kiel, Germany

## Abstract

In the title compound, C_11_H_8_ClN_3_, the azo group adopts a *trans* conformation and the dihedral angle between the six-membered rings is 15.47 (8)°.

## Related literature

For background to this work, see: Thies *et al.* (2010 ▶, 2011 ▶); Venkataramani *et al.* (2011 ▶). For the structure of a bis­(5-chloro-2-(phenyl­azo)pyridine)­dichloro–ruthenium(II) complex, see: Hansongnern *et al.* (2008 ▶).
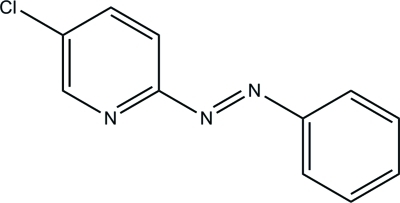


## Experimental

### 

#### Crystal data

C_11_H_8_ClN_3_
*M* *_r_* = 217.65
Monoclinic, 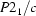

*a* = 6.1136 (2) Å
*b* = 9.0940 (4) Å
*c* = 18.6839 (8) Å
β = 91.459 (3)°
*V* = 1038.43 (7) Å^3^
*Z* = 4
Mo *K*α radiation
μ = 0.33 mm^−1^
*T* = 293 K
0.3 × 0.2 × 0.2 mm

#### Data collection

Stoe IPDS-2 diffractometer
19329 measured reflections
2818 independent reflections
2456 reflections with *I* > 2σ(*I*)
*R*_int_ = 0.028

#### Refinement

*R*[*F* ^2^ > 2σ(*F* ^2^)] = 0.044
*wR*(*F* ^2^) = 0.117
*S* = 1.15
2818 reflections
137 parameters
H-atom parameters constrained
Δρ_max_ = 0.22 e Å^−3^
Δρ_min_ = −0.17 e Å^−3^

### 

Data collection: *X-AREA* (Stoe & Cie, 2008 ▶); cell refinement: *X-AREA*; data reduction: *X-AREA*; program(s) used to solve structure: *SHELXS97* (Sheldrick, 2008 ▶); program(s) used to refine structure: *SHELXL97* (Sheldrick, 2008 ▶); molecular graphics: *XP* in *SHELXTL* (Sheldrick, 2008 ▶) and *DIAMOND* (Brandenburg, 2011 ▶); software used to prepare material for publication: *SHELXTL* (Sheldrick, 2008 ▶).

## Supplementary Material

Crystal structure: contains datablock(s) I, global. DOI: 10.1107/S1600536811047556/bt5703sup1.cif

Structure factors: contains datablock(s) I. DOI: 10.1107/S1600536811047556/bt5703Isup2.hkl

Supplementary material file. DOI: 10.1107/S1600536811047556/bt5703Isup3.cml

Additional supplementary materials:  crystallographic information; 3D view; checkCIF report

## Figures and Tables

**Fig. 1.**
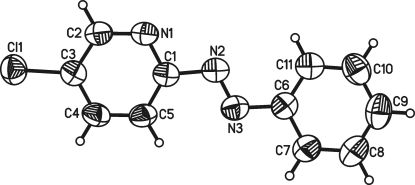
: Crystal structure of the title compound with labeling and displacement ellipsoids drawn at the 50% probability level.

**Fig. 2.**
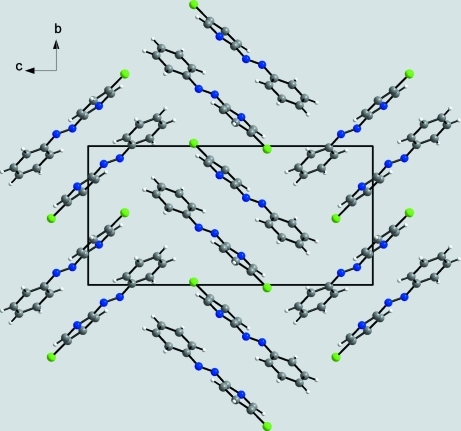
: Crystal structure of the title compound with view in the direction of the crystallographic c axis.

## supplementary crystallographic information

### Comment

We recently reported about a change of the spin state by association/dissociation of photodissociable ligands (PDL's) at square planar Ni(II) porphyrine complexes (Thies *et al.* 2010, Thies *et al.* 2011, Venkataramani *et al.*, 2011). Within this project the title compound, was obtained as an intermediate in the synthesis of 5-methoxy-2-phenylazopyridine which can be used as PDL. For the identification of this intermediate a structure determination was performed.

In the structure of the title compound, the 5-chloro-2-phenylazopyridine molecules, are not coplanar. Both 6-membered rings are twisted by 15.47 (8) °. The azo group is in a *trans* configuration and the torsion angle C1—N2—N3—C6 amounts to 178.5 (2) °). In the crystal structure the molecules exhibit a sandwich herringbone arrangement with neighbouring molecules stacked onto each other. The molecules are also linked by weak C—H···N interactions.

### Experimental

Synthesis of 5-Chloro-2-phenylazopyridine

A mixture of sodium hydroxide (12.0 ml of 25%), pyridine (8.00 ml) and 2-amino-5-chlorpyridine (15.6 mmol, 2.00 g) (Merck) was stirred at 80 °C. Nitrosobenzene (16.0 mmol, 1.71 g) dissolved in pyridine (60.0 ml) was added dropwise during a period of 45 min. The mixture was stirred for additional 30 min at 80 °C and stirred at RT for 72 h. The reaction mixture was extracted with toluene. The combined organic layer was dried over magnesium sulfate. After removal of the solvent, recrystallization with diethylether afforded red crystalls in 36% yield.

mp.: 84.5–87 °C

^1^H-NMR (600 MHz, 300 K, CDCl_3_, TMS): δ = 8.69 (d, ^4^J=2.4 Hz, 1H, 6-H), 8.04- 8.03 (m, 2H, 2`-H), 7.87 (dd, ^4^J=2.5 Hz, ^3^J=8.5 Hz, 1H, 4-H), 7.81 (d, ^3^J=8.5 Hz, 1H, 3-H), 7.53–7.56 (m, 3H, 3`-H, 4`-H) p.p.m.. ^13^C-NMR (150 MHz, 300 K, CDCl_3_, TMS): δ = 161.0 (C2), 152.3 (C10), 148.4 (C6), 138.1 (C4), 133.6 (C5), 132.5 (C40), 129.2 (C30), 123.7 (C20), 115.9 (C3) p.p.m.. MS (EI, 70 eV): m/*z*(%)= 217 (1) [*M*]^+^, 105 (89) [M—C_5_H_3_NCl]^+^. MS (CI, Isobutan): m/*z*(%)= 218 (100) [*M*+H]^+^. UV/Vis (Toluol): λ(_max_)(lg ε)= 315 nm (4.058), 448 nm (2.494).

### Refinement

The  H atoms were located in difference map but were positioned with idealized geometry with C—H = 0.93Å and refined with isotropic displacement parameters (*U*_iso_(H) = 1.2*U*_eq_(C)) using a riding model.

### Crystal data

**Table:**

C_11_H_8_ClN_3_	*F*(000) = 448
*M**_r_* = 217.65	*D*_x_ = 1.392 Mg m^−^^3^
Monoclinic, *P*2_1_/*c*	Mo *K*α radiation, λ = 0.71073 Å
*a* = 6.1136 (2) Å	Cell parameters from 23258 reflections
*b* = 9.0940 (4) Å	θ = 2.2–29.2°
*c* = 18.6839 (8) Å	µ = 0.33 mm^−^^1^
β = 91.459 (3)°	*T* = 293 K
*V* = 1038.43 (7) Å^3^	Block, colourless
*Z* = 4	0.3 × 0.2 × 0.2 mm

### Data collection

**Table:**

Stoe IPDS-2 diffractometer	2456 reflections with *I* > 2σ(*I*)
Radiation source: fine-focus sealed tube	*R*_int_ = 0.028
graphite	θ_max_ = 29.2°, θ_min_ = 2.2°
ω scans	*h* = −7→8
19329 measured reflections	*k* = −12→12
2818 independent reflections	*l* = −25→25

### Refinement

**Table:**

Refinement on *F*^2^	Secondary atom site location: difference Fourier map
Least-squares matrix: full	Hydrogen site location: inferred from neighbouring sites
*R*[*F*^2^ > 2σ(*F*^2^)] = 0.044	H-atom parameters constrained
*wR*(*F*^2^) = 0.117	*w* = 1/[σ^2^(*F*_o_^2^) + (0.0494*P*)^2^ + 0.1607*P*] where *P* = (*F*_o_^2^ + 2*F*_c_^2^)/3
*S* = 1.15	(Δ/σ)_max_ < 0.001
2818 reflections	Δρ_max_ = 0.22 e Å^−^^3^
137 parameters	Δρ_min_ = −0.17 e Å^−^^3^
0 restraints	Extinction correction: *SHELXL97* (Sheldrick, 2008), Fc^*^=kFc[1+0.001xFc^2^λ^3^/sin(2θ)]^-1/4^
Primary atom site location: structure-invariant direct methods	Extinction coefficient: 0.013 (2)

### Special details

**Table:**

Geometry. All e.s.d.'s (except the e.s.d. in the dihedral angle between two l.s. planes) are estimated using the full covariance matrix. The cell e.s.d.'s are taken into account individually in the estimation of e.s.d.'s in distances, angles and torsion angles; correlations between e.s.d.'s in cell parameters are only used when they are defined by crystal symmetry. An approximate (isotropic) treatment of cell e.s.d.'s is used for estimating e.s.d.'s involving l.s. planes.
Refinement. Refinement of *F*^2^ against ALL reflections. The weighted *R*-factor *wR* and goodness of fit *S* are based on *F*^2^, conventional *R*-factors *R* are based on *F*, with *F* set to zero for negative *F*^2^. The threshold expression of *F*^2^ > σ(*F*^2^) is used only for calculating *R*-factors(gt) *etc*. and is not relevant to the choice of reflections for refinement. *R*-factors based on *F*^2^ are statistically about twice as large as those based on *F*, and *R*- factors based on ALL data will be even larger.

### Fractional atomic coordinates and isotropic or equivalent isotropic displacement parameters (Å^2^)

**Table:**

	*x*	*y*	*z*	*U*_iso_*/*U*_eq_	
Cl1	0.82419 (7)	−0.02057 (5)	0.37109 (2)	0.07091 (17)	
C1	0.4709 (2)	0.27818 (16)	0.51393 (7)	0.0521 (3)	
C2	0.4706 (3)	0.12003 (19)	0.42028 (8)	0.0610 (4)	
H2	0.3950	0.0699	0.3840	0.073*	
N1	0.3601 (2)	0.20937 (16)	0.46186 (7)	0.0620 (3)	
C3	0.6925 (2)	0.09777 (16)	0.42821 (7)	0.0529 (3)	
C4	0.8081 (2)	0.17112 (19)	0.48142 (9)	0.0605 (4)	
H4	0.9585	0.1591	0.4872	0.073*	
C5	0.6949 (2)	0.26232 (18)	0.52564 (8)	0.0586 (4)	
H5	0.7666	0.3125	0.5627	0.070*	
N2	0.3334 (2)	0.37225 (14)	0.55510 (7)	0.0588 (3)	
N3	0.4247 (2)	0.41302 (15)	0.61134 (7)	0.0593 (3)	
C6	0.2969 (3)	0.51050 (16)	0.65384 (8)	0.0559 (3)	
C7	0.3993 (3)	0.5565 (2)	0.71665 (9)	0.0685 (4)	
H7	0.5377	0.5211	0.7292	0.082*	
C8	0.2973 (4)	0.6544 (2)	0.76071 (9)	0.0773 (5)	
H8	0.3672	0.6861	0.8027	0.093*	
C9	0.0916 (4)	0.7056 (2)	0.74256 (10)	0.0778 (5)	
H9	0.0224	0.7721	0.7723	0.093*	
C10	−0.0123 (3)	0.6581 (2)	0.68026 (11)	0.0750 (5)	
H10	−0.1523	0.6919	0.6686	0.090*	
C11	0.0890 (3)	0.56153 (19)	0.63543 (9)	0.0626 (4)	
H11	0.0192	0.5307	0.5932	0.075*	

### Atomic displacement parameters (Å^2^)

**Table:**

	*U*^11^	*U*^22^	*U*^33^	*U*^12^	*U*^13^	*U*^23^
Cl1	0.0752 (3)	0.0732 (3)	0.0646 (3)	0.0099 (2)	0.00810 (19)	−0.00282 (19)
C1	0.0511 (7)	0.0541 (7)	0.0511 (7)	−0.0032 (6)	−0.0005 (6)	0.0062 (6)
C2	0.0545 (8)	0.0704 (9)	0.0577 (8)	−0.0019 (7)	−0.0079 (6)	−0.0056 (7)
N1	0.0487 (6)	0.0749 (8)	0.0619 (7)	0.0003 (6)	−0.0077 (5)	−0.0061 (6)
C3	0.0534 (7)	0.0551 (7)	0.0502 (7)	−0.0004 (6)	0.0027 (6)	0.0077 (6)
C4	0.0429 (7)	0.0727 (9)	0.0658 (9)	−0.0039 (6)	−0.0015 (6)	0.0023 (7)
C5	0.0510 (7)	0.0670 (9)	0.0575 (8)	−0.0113 (6)	−0.0050 (6)	−0.0028 (7)
N2	0.0553 (7)	0.0622 (7)	0.0587 (7)	−0.0034 (6)	−0.0047 (5)	0.0004 (6)
N3	0.0580 (7)	0.0654 (7)	0.0540 (7)	−0.0035 (6)	−0.0044 (5)	0.0024 (6)
C6	0.0605 (8)	0.0544 (8)	0.0529 (7)	−0.0063 (6)	0.0053 (6)	0.0058 (6)
C7	0.0691 (10)	0.0797 (11)	0.0565 (8)	0.0001 (8)	−0.0019 (7)	−0.0023 (8)
C8	0.0968 (14)	0.0797 (12)	0.0554 (9)	−0.0021 (10)	0.0015 (9)	−0.0046 (8)
C9	0.1020 (14)	0.0649 (10)	0.0675 (10)	0.0085 (10)	0.0238 (10)	0.0046 (8)
C10	0.0705 (10)	0.0715 (11)	0.0835 (12)	0.0104 (8)	0.0104 (9)	0.0137 (9)
C11	0.0639 (9)	0.0614 (9)	0.0623 (9)	−0.0049 (7)	−0.0011 (7)	0.0069 (7)

### Geometric parameters (Å, °)

**Table:**

Cl1—C3	1.7288 (15)	N3—C6	1.435 (2)
C1—N1	1.3278 (19)	C6—C7	1.381 (2)
C1—C5	1.389 (2)	C6—C11	1.388 (2)
C1—N2	1.436 (2)	C7—C8	1.373 (3)
C2—N1	1.322 (2)	C7—H7	0.9300
C2—C3	1.376 (2)	C8—C9	1.375 (3)
C2—H2	0.9300	C8—H8	0.9300
C3—C4	1.377 (2)	C9—C10	1.381 (3)
C4—C5	1.370 (2)	C9—H9	0.9300
C4—H4	0.9300	C10—C11	1.372 (3)
C5—H5	0.9300	C10—H10	0.9300
N2—N3	1.2341 (17)	C11—H11	0.9300
			
N1—C1—C5	123.29 (15)	C7—C6—C11	120.09 (15)
N1—C1—N2	112.26 (13)	C7—C6—N3	114.62 (14)
C5—C1—N2	124.44 (13)	C11—C6—N3	125.27 (14)
N1—C2—C3	123.00 (14)	C8—C7—C6	120.23 (17)
N1—C2—H2	118.5	C8—C7—H7	119.9
C3—C2—H2	118.5	C6—C7—H7	119.9
C2—N1—C1	117.49 (13)	C7—C8—C9	119.86 (18)
C2—C3—C4	119.51 (14)	C7—C8—H8	120.1
C2—C3—Cl1	119.90 (12)	C9—C8—H8	120.1
C4—C3—Cl1	120.59 (12)	C8—C9—C10	120.02 (18)
C5—C4—C3	118.09 (14)	C8—C9—H9	120.0
C5—C4—H4	121.0	C10—C9—H9	120.0
C3—C4—H4	121.0	C11—C10—C9	120.62 (18)
C4—C5—C1	118.60 (14)	C11—C10—H10	119.7
C4—C5—H5	120.7	C9—C10—H10	119.7
C1—C5—H5	120.7	C10—C11—C6	119.17 (16)
N3—N2—C1	112.15 (13)	C10—C11—H11	120.4
N2—N3—C6	114.60 (13)	C6—C11—H11	120.4
